# SPARK: making ethical and societal tensions explicit in AI-supported precision medicine education

**DOI:** 10.1186/s12910-026-01484-6

**Published:** 2026-05-27

**Authors:** Viviam Solangeli Bermúdez Paiva, Anamika Chatterjee, Eirini Tsirvouli, Åsmund Flobak

**Affiliations:** 1 Department of Clinical and Molecular Medicine, Norwegian University of Science and Technology (NTNU), Trondheim, Norway; 2 Centre for Digital Life Norway, Norwegian University of Science and Technology (NTNU), Trondheim, Norway; 3 Department of Biology, Norwegian University of Science and Technology (NTNU), Trondheim, Norway; 4 The Cancer Clinic, St Olav’s University Hospital, Trondheim, Norway; 5 Department of Biotechnology and Nanomedicine, SINTEF Industry, Trondheim, Norway; 6 NEC OncoImmunity AS, Oslo, Norway

**Keywords:** Ethics deliberation, Precision medicine, Artificial intelligence, Medical education, Public engagement

## Abstract

**Background:**

Precision medicine is often framed in terms of technical innovation and clinical promise, while its broader ethical and societal implications receive less attention in medical training. Addressing these issues requires formats that allow participants to explore uncertainty, competing values, and governance challenges. This study examines SPARK, a scenario-based deliberative activity designed to support collective reflection on the systemic and value-laden dimensions of these technologies in educational settings.

**Methods:**

We analysed qualitative and descriptive-quantitative data from two university workshops in Norway involving students in medicine and bioethics. The SPARK activity uses structured scenarios, information cards, and issue prompts to guide small-group discussions of policy and implementation dilemmas related to artificial intelligence (AI)-supported precision medicine. The analysis focused on engagement processes, examining how participants articulated trade-offs, uncertainties, and competing priorities during deliberation.

**Results:**

Participants initially framed precision medicine primarily in technical and individualised terms. During the activity, discussions expanded to include ethical and societal considerations such as fairness, responsibility, and resource allocation. The deliberative structure enabled participants to articulate trade-offs and negotiate competing priorities while moving between individual and group decision stages. Rather than resolving disagreements, the activity made underlying tensions explicit and supported discussion of competing ethical considerations within a time-limited educational setting.

**Conclusions:**

SPARK demonstrates how lightweight deliberative formats can support structured ethical reflection on emerging medical technologies in educational contexts. By making tensions visible and negotiable within group discussion, such formats may complement existing approaches to ethics education and help create space for engagement with the broader implications of AI-supported precision medicine.

**Graphical Abstract:**

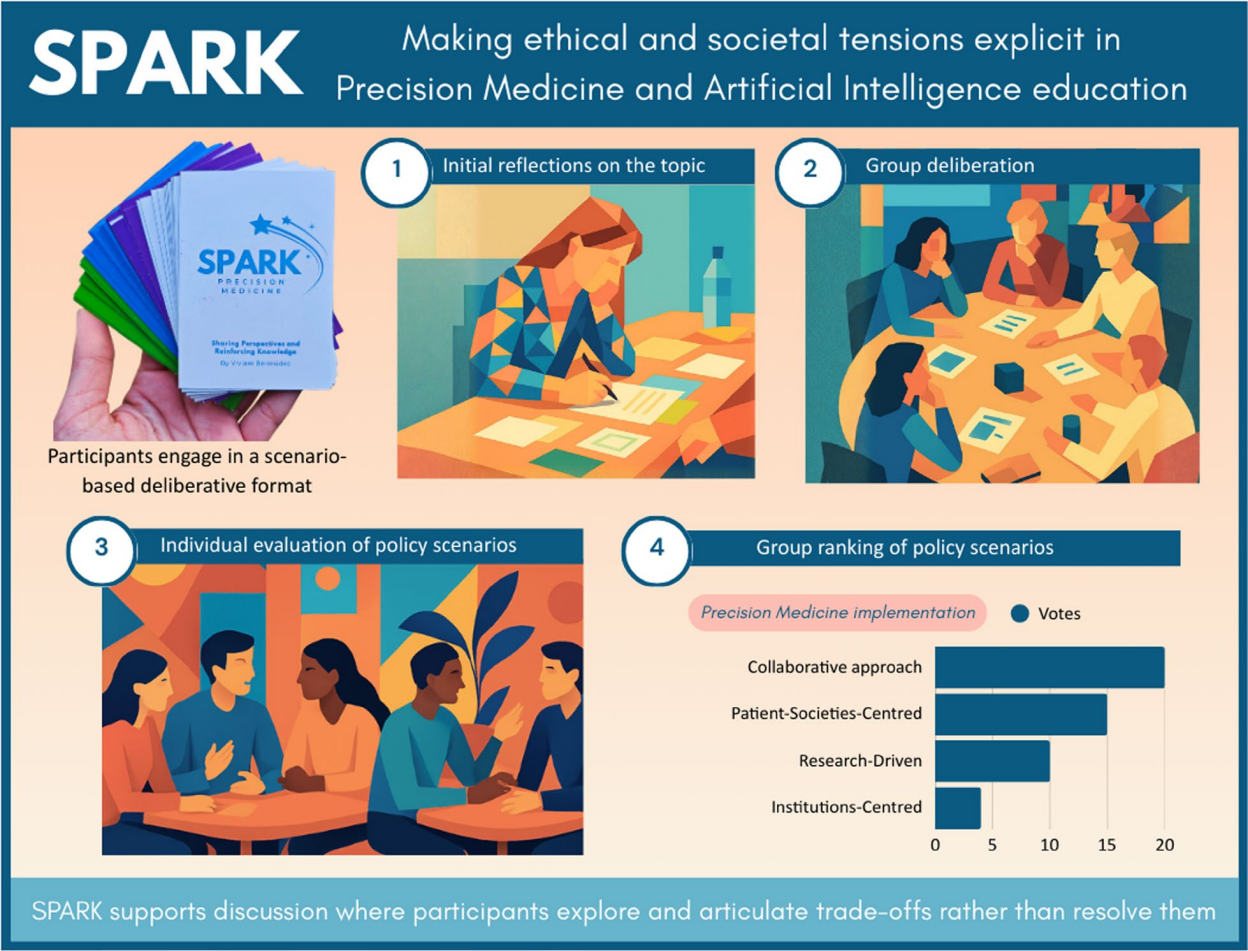

## Background

Problem-based learning (PBL) and team-based learning (TBL) are widely adopted approaches for student-centred learning in medical education. PBL supports small-group work on complex problems and has played a vital role in Norwegian medical education reform, particularly through its early implementation in Trondheim, aiming to integrate theory, clinical reasoning, and professional skills [1, 2]. However, PBL is resource-intensive, which hampers scalability, consistency, and alignment with institutional frameworks [3, 4]. TBL was developed to maintain collaborative problem-solving while enhancing scalability [5]. Although TBL enables fewer facilitators to manage larger groups, it relies heavily on pre-class preparation and structured assessment, and differences in student readiness can impact participation [6]. The practical challenges are particularly evident when teaching topics involving ethical and societal dilemmas in medicine, where meaningful engagement requires space for discussion and reflection, but managing large cohorts makes implementation difficult. Understanding how future healthcare professionals reason about the ethical implications of emerging medical technologies has therefore become an important concern in contemporary medical ethics.

Among contemporary medical ethics topics, emerging technologies such as precision medicine (PM) and artificial intelligence (AI) pose unique challenges that extend beyond clinical decision-making. These technologies are increasingly shaping research and clinical practice, and AI is considered a key enabling component within PM. By tailoring diagnosis and treatment to each individual’s biological traits, PM aims to improve clinical outcomes and more efficiently use healthcare resources [7]. Recent advances in biotechnology and AI allow for detailed characterisation of individual variability, leading to a deeper understanding of disease risk and progression [8]. This shift enables more targeted and precise interventions tailored to each patient’s specific characteristics.

At the same time, biotechnology developments raise questions about data use and misuse, equity, and trust [7, 9, 10], which aren’t easily addressed through conventional teaching methods. Engaging with such issues requires space for discussion and reflection, rather than convergence on a single correct answer [11]. From an educational perspective, this aligns with PBL and TLB principles that promote collective reasoning and engagement [5, 12]. However, providing discussion-oriented learning experiences at scale remains challenging, owing to the need for trained facilitators, advanced preparation, and structured readiness mechanisms [1, 6]. Emerging hybrid and digitally supported adaptations seek to address these limitations, aiming to preserve core learning principles while improving feasibility and scalability [4, 13, 14]. Therefore, it is essential to develop structured activities that not only prioritise the ethical and societal dimensions of emerging technologies but also seamlessly integrate into today’s educational landscape.

SPARK (Sharing Perspectives And Reinforcing Knowledge) was developed in response to this need as a structured, scenario-based learning activity designed to support discussion of ethical and societal dilemmas related to PM and AI. It incorporates PBL and TBL principles, such as engagement with concrete issues, small-group discussions, and structured in-session tasks, while limiting facilitation and preparation demands. Utilising the PlayDecide framework (available at: https://playdecide.eu/en/about*)*, SPARK employs story, information, and issue cards to facilitate discussions on complex topics. Rather than seeking consensus, it encourages participants to articulate concerns, negotiate trade-offs, and consider multiple perspectives. Unlike traditional PBL tutorials, SPARK does not require one facilitator per group, and unlike TBL, it does not rely on advance preparation or readiness assurance testing. A small number of facilitators can oversee multiple tables, enabling 40–80 participants to engage simultaneously while maintaining core dialogical elements.

In this paper, we introduce SPARK, a structured deliberative learning activity aimed at fostering discussions around the ethical and societal dimensions of PM in contexts where AI supports PM rather than dominates. Based on qualitative and descriptive-quantitative data collected from two workshop sessions at Norwegian universities, this study addresses two related questions. First, how can SPARK serve as a scalable and practical extension of student-active approaches in education settings? Second, what insights and reflections arise when students are given space to explore the ethical, societal, and system-level dimensions of PM? The study highlights how structured deliberation influences engagement with these topics, revealing the variety of reflections and tensions that may emerge. Engaging in such discussions is vital for cultivating ethical awareness, perspective-taking, and reflective skills in professional education. This paper ultimately offers a transferable and scalable approach for incorporating ethical reflection into contemporary educational settings, primarily targeting interpretive dimensions of learning.

## Methods

### Study design

The data analysed in this study were collected during two SPARK workshop sessions conducted in educational settings in two Norwegian universities in 2025. The study employed a mixed-methods, exploratory design based on structured, scenario-based workshops. Ethical and societal aspects of precision medicine (PM) in cancer research and the application of artificial intelligence (AI) in PM medical settings were the focus of the workshops. The SPARK workshop is reported in detail using the TIDieR checklist (Supplementary material S1), adapted from [15].

Quantitative and qualitative data were collected concurrently during the workshop sessions, including individual inputs, group-level prioritisation, and written reflections. Assessment of learning outcomes, behavioural changes, or consensus formation was outside the scope of the present study. The study was descriptive and focused on participants’ engagement with the topic during the workshop.

### Participants and settings

A total of 101 participants took part in two analysed SPARK sessions. The first session included 33 interdisciplinary graduate students enrolled in a bioethics course. The second session included 68 first-year medical students. No demographic data beyond course affiliation were collected. Two additional SPARK sessions were conducted prior to these workshops to pilot and refine the workshop materials and format; however, they are not included in the analysis presented here.

Each workshop session was facilitated by two or three facilitators. Facilitators varied across sessions but adhered to the exact procedural instructions and did not guide the discussion content. Across the two analysed sessions, the same types of data were collected, including pre- and post-workshop polls, individual voting, group prioritisation, written placemat notes, and post-workshop takeaway responses.

Participation in the SPARK workshops was voluntary and integrated into scheduled teaching sessions. In the medical student cohort, around 200 students were enrolled in the course, with an initial poll indicating that 95 were interested in participating in the workshop. Ultimately, on the day of the workshop, 68 students engaged actively in the session. In the bioethics student cohort, approximately 55 students were enrolled in the course, of whom 33 participated in the workshop activities; however, no prior poll was conducted to assess interest in participating in this cohort. Participation varied across workshop activities, as engagement in digital inputs and written materials was voluntary (see Data analysis and Ethical considerations sections). The figures reported in this study refer specifically to participation in the digital components of the sessions (see Workshop activities and data sources section).

### The SPARK workshop

The SPARK workshop was developed using the PlayDecide framework (available at: https://playdecide.eu/en/about*)*, a card-based group discussion format. While retaining the core principle of facilitated small-group discussion, SPARK was adapted to the topic of data-driven and AI-driven PM in cancer care, with a focus on general understanding and student engagement.

**Fig. 1:**
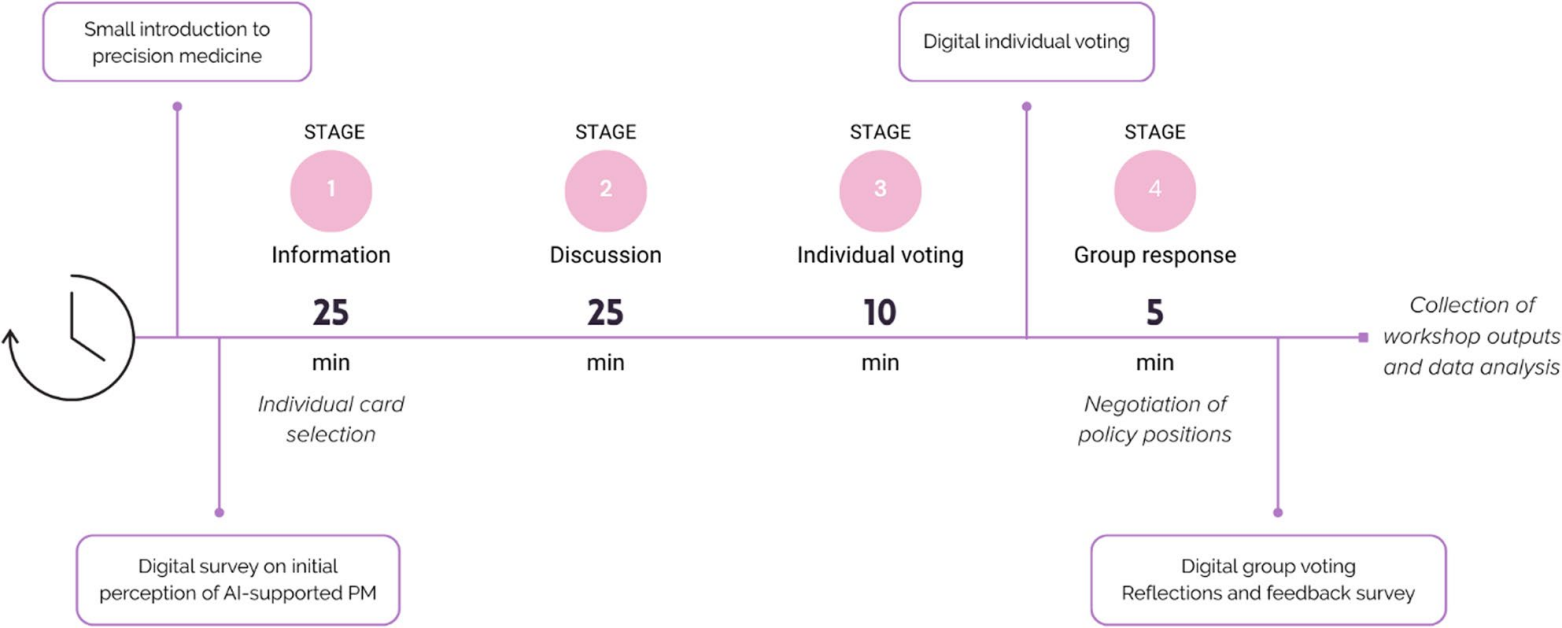
Workshop structured activities. The workshop lasts 50–120 min, starting with a brief PM intro and individual digital inputs via polls. It then proceeds through four stages: Stage 1 - Information (25 min), where participants review cards; Stage 2 - Discussion (25 min) with small group talks (4–8 people); Stage 3 - Individual voting (10 min) to assess scenarios for PM implementation; and Stage 4 - Group response (5 min) to prioritise a scenario. The session ends with reflections and feedback via digital polls

The two workshops were conducted in small groups of four to eight participants, resulting in 11 groups in the medical student session and 8 in the bioethics student session. Each session lasted 50 to 120 mins and followed a semi-structured sequence of activities, including individual reflection, group discussion, individual voting, and collective prioritisation (Fig. 1). Facilitators introduced the activity and provided procedural instructions, but did not deliver formal teaching or guide the content of discussions. Their role was limited to supporting the session’s flow and ensuring that all participants had the opportunity to contribute.

### SPARK cards

The content of the SPARK cards was developed specifically for this study. Source material was drawn exclusively from publicly available research articles and media coverage on PM, data-driven and AI-driven decision-making in cancer care. A complete description of sources and content is provided in Supplementary material S2. After reviewing these sources, key concepts and themes were extracted and translated into concise card texts. The cards were designed to represent a range of perspectives and tensions in a format suitable for structured group discussion. Technical language was simplified to ensure accessibility for participants with diverse backgrounds, while preserving the substantive issues and perspectives reflected in the source material.

Following an initial drafting phase, the card content underwent an iterative review process involving three colleagues with expertise in related fields. Feedback from this review was used to refine wording, clarify concepts, and improve balance across perspectives prior to use in the workshops. The number and mix of cards were determined pragmatically to provide sufficient breadth of perspectives while remaining manageable within a time-limited workshop setting. The cards’ content is available in Supplementary material S2 under an Attribution-ShareAlike 4.0 International (CC BY-SA 4.0) license.

#### Card type

**Fig. 2:**
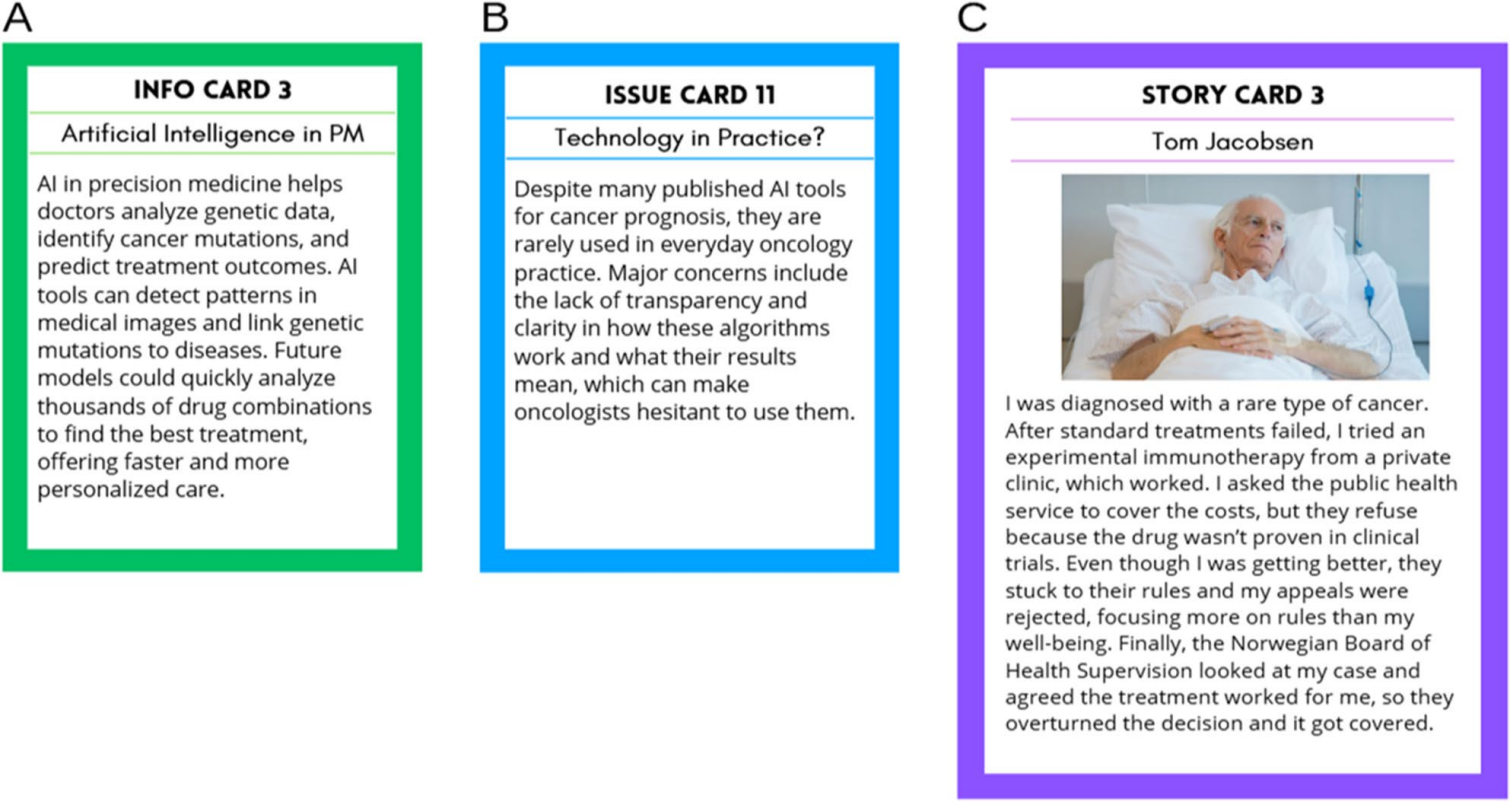
SPARK card types. **A** Information cards: present factual or contextual information related to PM, such as research practices, data use, or clinical implementation. **B** Issue cards: highlighted ethical or societal questions and tensions associated with PM, including trade-offs among competing values. **C** Story cards: presented short, fictionalised scenarios inspired by real-world contexts to support perspective-taking and discussion of potential impacts on patients, professionals, or institutions

The SPARK workshop used three distinct types of cards, each serving a specific role in structuring the discussion (Fig. 2). All workshop sessions included cards from each category. Depending on session duration and group size, either the whole card set or a reduced subset was used.

### Workshop activities and data sources

During the workshop, participants were invited to read and discuss the cards collectively within their groups, using them as shared reference points to structure the discussion (Fig. 1). In addition to discussion, the workshop included several structured activities designed to capture individual and collective responses. The polling tool Mentimeter was used to collect responses digitally (https://www.mentimeter.com/).

Participants completed an initial free-text poll on the question “What comes to your mind when talking about Precision Medicine?” before and after engaging with the cards. Responses to the open-ended question were aggregated and visualised as word clouds, which served both as a real-time engagement tool during the workshop and as a descriptive representation of commonly mentioned terms before and after the activity.

In the initial stage, participants were instructed to select one card of each type (information, issue, and story) from the shared pool of cards. Cards were selected based on what participants found interesting, novel, or relevant and placed on the individual placemats. Participants then took turns presenting their selected cards to the group before the discussion phase. Selected cards remained available throughout the activity and were not returned to the pool. On the discussion stage, participants exchanged opinions about the cards’ content and could use them as reference points. After the discussion stage, participants individually rated their level of agreement with a set of predefined PM scenarios (Table 1) using the digital polling tool. 56 responses were collected in the medical student session and 33 in the bioethics session. Small groups were then asked to collectively prioritise one scenario through discussion and group-level voting, resulting in 11 group votes in the medical student session and 8 in the bioethics session.

**Table 1:** Predefined scenarios related to precision medicine research and implementation

Policy Scenario	Description
1. Patient-Societies-Centered	Cancer patient societies guide precision medicine decisions, both implementation in healthcare systems and new research. Public needs are prioritised and networks are established to make new technologies transparent and accessible to patients. Individuals are actively involved in precision medicine development.
2. Collaborative Approach	Decisions on precision medicine research and implementation should be made through collaboration among doctors, researchers, and policymakers, ensuring diverse perspectives and fair access for all. Networks should be built to share knowledge and data across institutions and borders.
3. Research-Driven	The primary focus of precision medicine should be advancing scientific research. The health system must prioritise funding and infrastructure to support the latest research. Healthcare implementation should be based on proven scientific results, even if that means patients have less influence on individual treatment choices.
4. Institutions-Centered	Precision medicine should be managed and directed by healthcare authorities, without over-relying on patient or researcher influence. Hospitals decide how it is implemented, ensuring that care is standardised and efficient. They focus on building AI systems, data management, and professional training to integrate precision medicine into routine care.

The full description of each policy scenario was provided to participants as part of the individual placemat material used during the workshop. Scenarios were presented in written form and remained available throughout the activity. This ensured that participants could refer to the content when making both individual and group decisions. Placemat content is available in Supplementary material S3, including a printing version.

Each participant received a placemat with additional space for note-taking. Placemat notes were optional and open-ended, allowing participants to record topics discussed, personal comments, and opinions. All placemats were collected at the end of the session (see Fig. 1); some contained written notes, while others were left blank. Because these notes were voluntary and unevenly distributed across small groups, they were not subjected to systematic coding or frequency analysis; instead, they were used descriptively to illustrate and triangulate tensions identified through card-based analysis (see Tensions and card uptake analysis section).

Finally, participants were invited to provide short digital reflective responses to the prompt *“What stuck with you from this discussion?*” and “*Write one impactful takeaway from this workshop*”, along with general feedback on the activity. The question *“What stuck with you from this discussion?”* was administered only in the medical student session and is therefore reported for that session alone (Supplementary material S4).

Responses were automatically grouped into thematic categories by the digital polling tool (Supplementary material S4). These groupings were used as a descriptive aid; no additional systematic recording or quantitative reclassification was performed. Selected uncategorized responses were interpreted descriptively when clearly aligned with tensions identified elsewhere.

### Data analysis

Quantitative data were derived from individual voting responses and group-level prioritisation activities conducted during the workshops. Individual responses to predefined scenarios were collected using the digital polling tool and summarised using descriptive statistics. These data were used to examine overall patterns of agreement across scenarios and to identify variation in individual responses within the participant cohort.

Group-level data were generated through collective prioritisation exercises in which each group selected a single scenario after discussion. Group selections were compared descriptively with the distribution of individual responses to explore areas of convergence and divergence between individual judgments and collective outcomes. No inferential statistical analyses were conducted, and findings are reported descriptively.

Qualitative data included written placemat notes generated during group discussions, free-text responses to pre- and post-workshop polls, and short written responses to the prompt “Write one impactful takeaway from this workshop.” All qualitative materials were anonymised before analysis.

#### Tensions and card uptake analysis

As part of the post-workshop analysis, issue and story cards were grouped by the investigators into six themes that cover common ethical challenges in data-driven health care (Table 2). The thematic grouping was conducted after data collection and was not used during the workshop or visible to participants. Instead, theme grouping serves as an analytical framework to describe patterns in how different issues were taken up during group deliberation. Information cards were used to provide background context during the activity, but were excluded from this tension analysis.

**Table 2:** Overview of the tension themes of the SPARK card set. The table summarises six overarching themes used to organise the ethical and societal issues represented in the cards, along with brief definitions and the number of cards associated with each theme

Tension theme	Definition	Number of cards
Promise vs limits in practice	High expectations for PM and AI contrasted with uneven effectiveness, delays, failures, and unmet hopes in real-world use	3
Personal benefit vs system constraints	Potential benefits for individual patients contrasted with costs, resource limitations, reimbursement rules, and health system capacity	5
Data use vs loss of control	Use of genetic and health data contrasted with loss of control over future use and implications for individuals and their families	3
Inclusion vs exclusion	Questions about who is represented, protected, or benefited by PM versus who may be left out or disadvantaged	3
Understanding vs complexity	Increased access to information and AI-based tools contrasted with challenges in understanding, interpreting, and acting on complex outputs	4
Innovation vs responsibility	Pressures to advance technological innovation contrast with responsibility for accuracy, bias, oversight, and broader societal consequences	2

To describe which tensions were taken up during group deliberation, card selections were analysed at the level of tension themes (Supplementary material S5). Card selection was treated as an indicator of which tensions were initially taken up during deliberation within each group, and not as a measure of importance, agreement, or consensus. To account for differences in the number of cards addressing each theme, selections were normalised by the number of available cards per theme. Uptake ratios were calculated for each small group by dividing the number of selected cards associated with a given tension by the total number of cards representing that tension.

Uptake patterns were examined at the table level and aggregated across tables to characterise overall engagement patterns. Participants recorded their selections on placemats, though some remained blank. Placemat data were available for 8 out of 11 groups in the medical student session, and analysis was limited to 7 groups with more than four participants with identifiable selections. Uptake ratios are reported descriptively, with mean values across eligible groups. This analysis was conducted for the medical student cohort only, as no groups in the bioethics session met the inclusion criteria (four participants with identifiable selections).

### Ethical considerations

Participation in the SPARK workshop was voluntary. Participants were informed that their anonymised responses and written materials could be used for research purposes. No personally identifiable information was collected, and all data were analysed in aggregate. The workshop was conducted as part of educational and engagement activities and did not involve interventions, assessments, or evaluations of individual participants. Based on the nature of the activity and local institutional guidelines, formal ethical approval was not required.

## Results

### Perceptions on precision medicine

To examine how participants’ perceptions of precision medicine (PM) were articulated before and after the workshop, participants were asked to reflect on PM at two time points. Responses were collected via an open-ended digital poll and displayed as word clouds, highlighting the most frequently mentioned terms. These visualisations illustrate the range of associations made by participants but do not indicate frequency, context, or individual-level variation. At the beginning of the session, first-year medical students (*n* = 68) and bioethics students (*n* = 33) responded to the question “What comes to your mind when talking about PM?”.

**Fig. 3:**
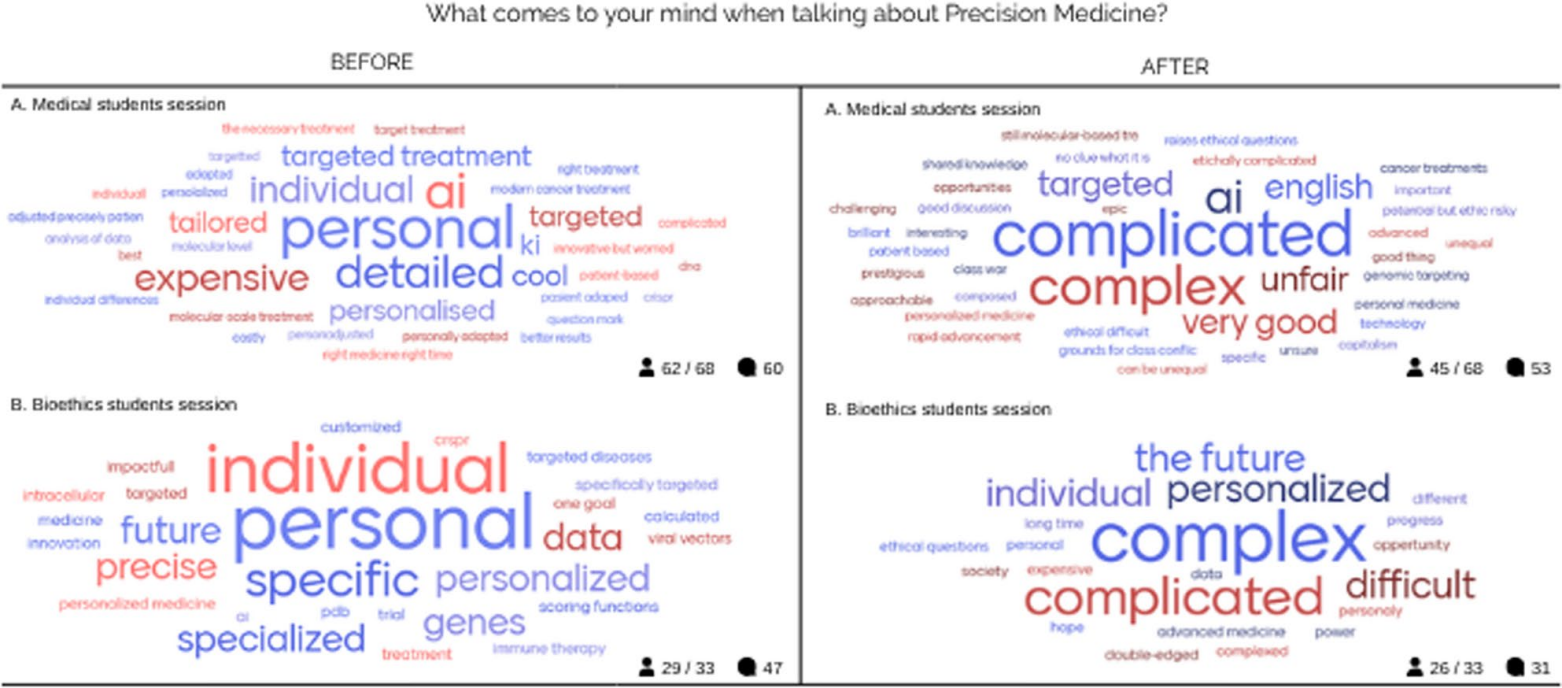
Reflections on PM before and after SPARK. Word clouds visualising responses to the prompt “Precision medicine in one word” from (**a**) first-year medical students and (**b**) bioethics students, before the workshop (left panel), and after the workshop (right panel). Larger words indicate more frequently used terms

Before deliberation, participants across both cohorts predominantly framed PM in terms of personalisation and individualised treatment, using terms such as *“personal”* (mentioned 14 times across sessions) and *“individual”* (mentioned 9 times across sessions) (Fig. 3; Table 3). PM was commonly associated with targeted treatments and biomedical or technological elements, including genes, data, and molecular-level interventions. Medical students tended to use more outcome-oriented language (e.g., *“cool*,*” “future*,*” and “better results”*), while bioethics students more frequently referred to technical and research-oriented concepts (e.g., *“genes*,*” “CRISPR*,*” “viral vectors*,*”* and *“trials”*). Across both cohorts, ethical, societal, and systemic considerations—such as equity, access, or data governance—were largely absent from these initial responses.

**Table 3:** Summarised the most frequently mentioned terms underlying the word-cloud visualisations

Cohort	Stage	Most frequent terms (counts)
Medical	Before	personal (7), AI (5), detailed (4), individual (3)
Medical	After	complicated (5), complex (4), AI (3)
Bioethics	Before	personal (7), individual (6), specific (3)
Bioethics	After	complex (5), complicated (3), difficult (2)

After the activity, participants articulated a more complex and ethically situated understanding of PM. Word-cloud responses highlighted language emphasising complexity and challenge (e.g., *“complex”,* mentioned 9 times, *and “complicated”* mentioned 8 times across sessions), alongside ethical and societal concerns such as *“unfair*,*” “unequal*,*”* and *“power.”* These terms appeared alongside references to potential benefits and future opportunities, including *“important*,*” “opportunities*,*”* and *“the future”.*

This shift in framing was further reflected in open-ended post-discussion responses from the medical student session (42 responses) to the question “*What stuck with you from this discussion?”.* Participants frequently articulated concerns related to equity, prioritisation, and resource allocation, noting risks of increased inequality (e.g. *“It may lead to bigger differences in treatment given due to costs”*) and questioning how limited resources should be allocated (e.g. *“It is difficult to decide the value vs the cost*,* and it can be unfair as to who can get it”*). Other reflections situated these concerns within broader societal and global contexts, while some participants highlighted the pace of technological development and the need for ethical reflection to keep up with innovation. Full reflection responses are available in Supplementary material S4 and S6.

Taken together, these before-and-after responses suggest a broader and more ethically situated range of associations with PM following the activity. These patterns illustrate how structured deliberation surfaces additional dimensions of reflection, including ethical trade-offs, uncertainty, and societal implications alongside potential benefits.

### Concerns and tensions were actively taken up during deliberation

**Fig. 4:**
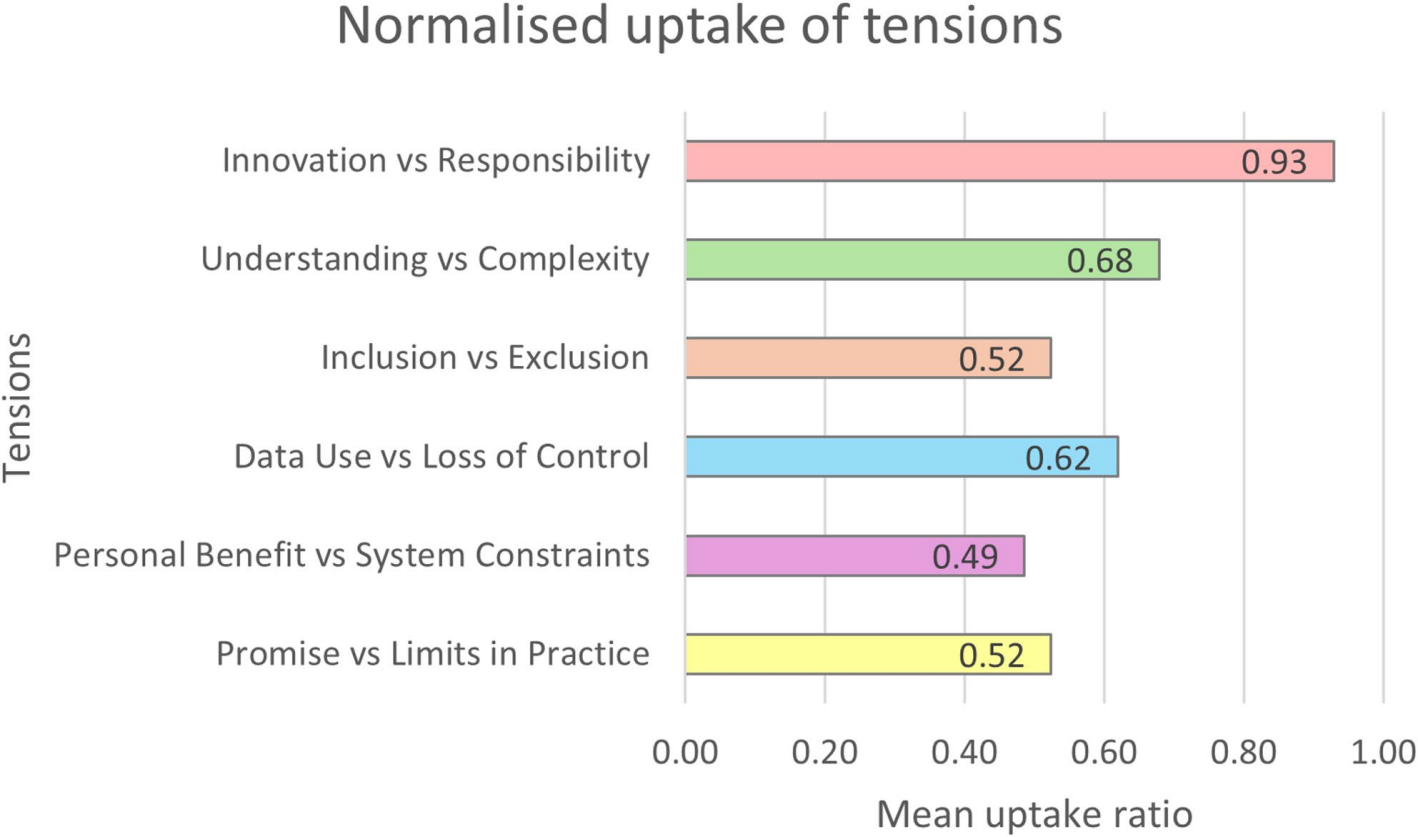
Normalised uptake of tensions during group deliberation. Bars represent the mean uptake ratio of tensions across eligible groups (n = 7), calculated as the number of times cards associated with each tension were selected divided by the number of available cards representing that tension. Normalisation accounts for the unequal representation of tensions in the card set. Uptake reflects participants’ engagement with the ethical issues available during the activity and should not be interpreted as importance, agreement, or consensus

To examine which tensions were taken up during group deliberation, we analysed participants’ card selections during the SPARK sessions with medical students. Tensions refer to recurring dilemmas embedded in the SPARK materials. For analytical purposes, the investigators grouped issue and story cards into six post-workshop tension themes (Table 2). Card selections were normalised and analysed descriptively. Figure 4 shows the mean uptake ratio across eligible groups (*n* = 7, see Methods for inclusion criteria). Additionally, participants were invited to make voluntary notes on placemats during the activity, including discussions and personal comments. Since participation was optional, these notes served a descriptive function, showing that tensions identified through card-based analysis were addressed in discussion.

Innovation vs Responsibility was the most frequently taken up tension, despite being represented by only two cards in the deck (normalised uptake mean = 0.93). This uptake indicates that issues related to responsibility and accountability in AI-supported PM were repeatedly made available for discussion. In written reflections, some participants articulated concerns about bias in AI-based research (*“Difficult to identify bias in AI and research”*) and questioned the implications of implementing new technologies (*“How it affects society and how important it is to be aware of it before implementing the technology”*).

Understanding vs Complexity, which reflected challenges related to interpreting and communicating complex genetic and algorithmic information, was also frequently selected (mean = 0.68). Participants articulated concerns connected to this tension, including the need for clearer language and greater technological literacy in clinical contexts. For example, participants questioned whether *“the importance of simple language”* is adequately addressed when discussing advanced technologies, asked *“do doctors need an IT education as well because of the new technology?”*, and reflected on personal preparedness, noting *“I need to develop my technological sense”.*

Several other tensions were also taken up across groups through card selection, indicating that a range of dilemmas was made available for discussion during the activity. Tensions related to Data Use vs Loss of Control were reflected in written comments concerning consent, transparency, and participant awareness in research contexts. For example, some participants noted that *“Patient has to know the risks and benefits of participating in research*,*”* while others questioned established practices by asking, *“Is written consent always the key in science?”.*

Tensions related to Personal Benefit vs System Constraints and Inclusion vs Exclusion were illustrated through reflections on cost, fairness, and prioritisation. Participants raised questions about the societal implications of investing in highly personalised treatments, as reflected in comments such as *“Expensive research on expensive medicines – is it worth it?”* and *“Norwegian perspective is skewed: Malaria vaccine is needed*,* but we spend millions on specific cancers.”* Issues of access and genetic rarity were also touched upon, with participants asking whether personalised approaches might introduce new forms of inequality, for instance: *“Should someone get treated with an expensive medicine just because their genetics are rare?”* and *“personal medicine can lead to that people don’t get insurance because they have a specific gene.”* These reflections were sometimes summarised as broader trade-offs between *“individual benefits vs greater good.”*

Finally, some written comments related to Promise vs Limits in Practice expressed uncertainty about expectations surrounding PM, including questions about potential overpromising, such as: *“Can precision medicine give false hope?”.* Together with the rest of the comments, these patterns reflect how participants selectively activated tensions during deliberation with freedom in card choice, articulating and negotiating ethical tensions within different groups.

### Individual ratings and group prioritisation of scenarios

As part of the SPARK activity, participants were asked to engage with four scenarios describing different approaches to implementing PM (Table 1). These scenarios were intentionally designed to surface contested priorities (e.g., collaboration, institutional control, research focus, and patient–society considerations). Participants first rated their individual agreement with each scenario and then, following group discussion, collectively prioritised them, allowing comparison between individual positioning and group-level decisions after deliberation.

**Fig. 5:**
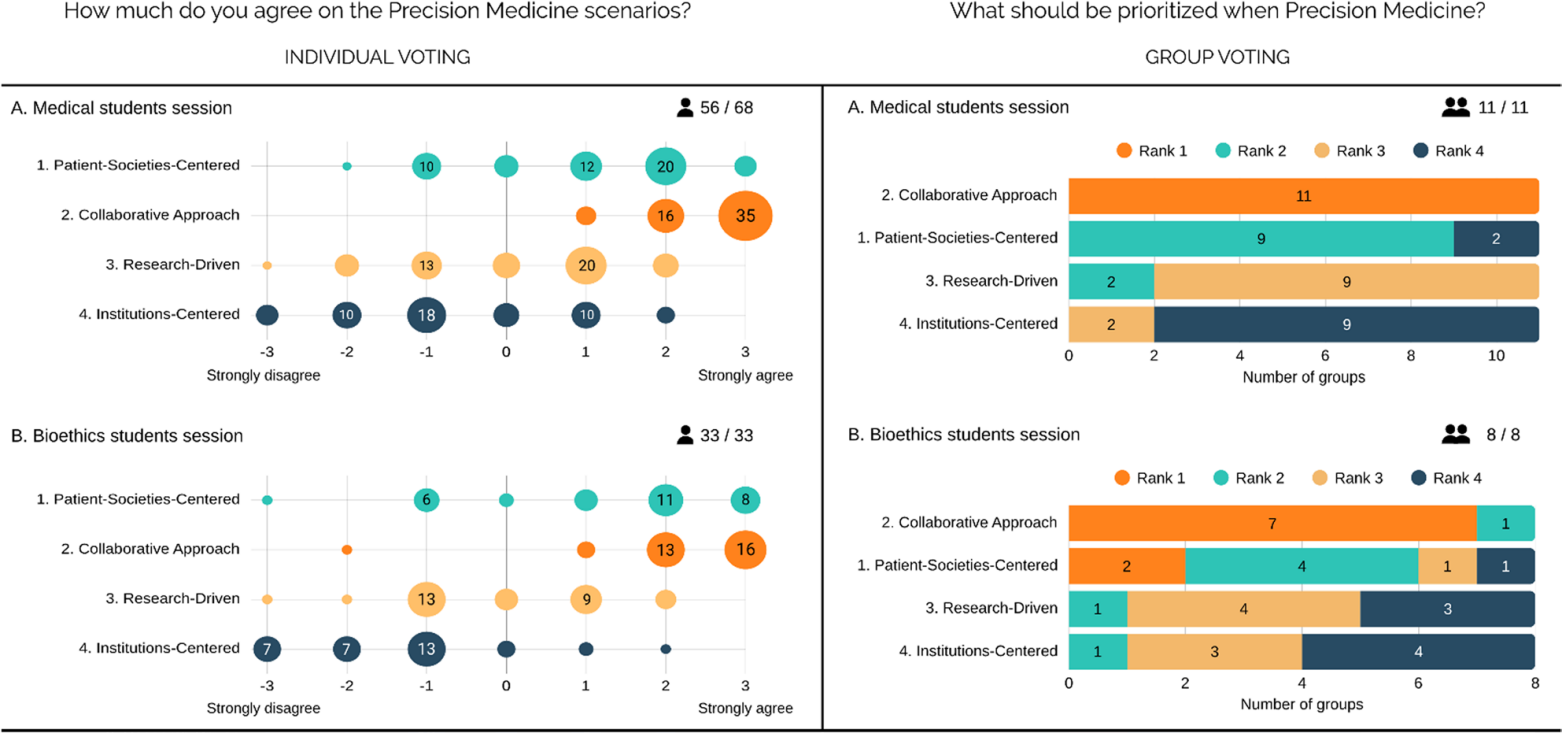
Individual ratings and group prioritisation of precision medicine implementation scenarios. Left panel: Distribution of individual agreement ratings for four PM implementation scenarios (patient–society-centered, collaborative, research-driven, and institution-centred). Each circle represents the number of participants selecting a given response level, with circle size and embedded values indicating counts. Ratings reflect participants’ individual positioning before group prioritisation and illustrate the diversity of attitudes within each cohort. Right panel: Group-level prioritisation of the same PM scenarios. Bars represent the number of groups assigning each ranking position (Rank 1–4), with counts displayed within segments. In both panels, results are shown separately for the medical student (**a**) and bioethics student (**b**) sessions

Across both the medical (*n* = 56) and bioethics students (*n* = 33) who participated in the digital poll, individual ratings showed variation in the degree of agreement across the four scenarios (Fig. 5, left panel), reflecting that participants could express partial or parallel support or agreement for multiple approaches rather than a single exclusive preference. Overall, the collaborative approach received the strongest individual support, with 35 “strongly agree” votes in the medical student session and 16 votes in the bioethics student session, while the institution-centred scenario was generally rated less favourably. Ratings for the patient–society-centred and research-driven scenarios were more dispersed, indicating heterogeneity in individual attitudes rather than uniform consensus within the groups.

After discussion, group prioritisation resulted in clearer collective patterns (Fig. 5, right panel). Across all groups (11 in the medical student session and 8 in the bioethics session), a consistent pattern emerged: groups consistently ranked the collaborative approach as the highest priority, while the institution-centred scenario was most often ranked lowest. Rather than indicating a shift in preferences, this alignment suggests that group deliberation consolidated an already salient orientation toward collaboration. The requirement to arrive at a shared ranking nonetheless involved discussion and negotiation, particularly in ordering the remaining scenarios, providing context for the post-deliberation reflections examined in the following section.

### Post-workshop reflections on the format

To examine how participants experienced SPARK as a learning and discussion format, participants were invited at the end of the workshop to respond individually to the prompt *“Write one impactful takeaway from this workshop”* via digital poll. This prompt generated 33 responses in the medical student session and 10 responses in the bioethics student session. Responses were short, open-ended, and automatically grouped by the digital polling tool into thematic categories (Supplementary material S4).

Across both sessions, participants most frequently highlighted the workshop’s interactive, discussion-based nature. Many reflections emphasised the value of discussing ethical issues with peers and hearing different perspectives, describing the activity as “*engaging*,” “*fun*,” and a “*good way to discuss ethics with others.*” Several participants noted that the format supported reflection and perspective-taking, for example, by enabling “different perspectives and opinions I didn’t think about before” and offering “a great way to explore multiple perspectives.” Alongside these reflections, some participants offered more critical or reflexive feedback, including a desire for more background information prior to the session, challenges in discussing complex topics in English, and limited time for discussion. Taken together, these comments suggest that participants experienced SPARK as a participatory space for ethical dialogue, while also highlighting practical considerations for its implementation in future educational contexts.

## Discussion

This study addresses two related questions: how SPARK functions as a structured, scenario-based deliberative activity in educational settings, and what kinds of reflections emerge when students are given space to engage with the broader implications of artificial intelligence (AI)-supported precision medicine (PM). The emphasis of this discussion is on the engagement processes: which issues are addressed, how trade-offs are managed, and how individual and collective reasoning develop within a discussion format that is intentionally structured by materials and prompts.

A pattern emerges across the results: initially, participants framed PM through technical and individualised terms, overlooking broader issues such as fairness, responsibility, or resource allocation. During SPARK, these considerations were more explicitly addressed, with cards and policy scenarios serving as common references for comparison and contrast across viewpoints. Participants articulated issues, made trade-offs explicit, and negotiated among considerations, rather than changing individual perspectives. Ethical and societal implications of PM become central topics of discussion, topics often sidelined in traditional lectures. In this way, SPARK fosters engagement that is less readily achieved through conventional teaching formats, addressing the need for structured reflection on the societal impact of emerging medical technologies in today’s educational settings [11, 13].

The design of SPARK seemed to shape both how discussions were organised and what issues were brought into focus. Rather than offering a neutral discussion space, this structure intentionally foregrounds diverse and sometimes conflicting perspectives, ensuring that key ethical tensions are brought into the discussion. Repeated engagement with different tensions suggests that participants used the activity structure to explore the interconnections among issues, rather than to decide which concern should take precedence. In this way, the value of the activity lies not simply in generating discussion but in providing opportunities to articulate, confront, and negotiate ethical tensions that might otherwise remain implicit or unexamined.

The contrast between individual ratings and group prioritisation reflects the different forms of reasoning involved at each stage of the activity. During individual voting, participants could express graded agreement with multiple PM policy scenarios at once. In contrast, group prioritisation required participants to collectively rank the scenarios and agree on a single shared ordering. Importantly, reaching a shared ranking did not imply uniform views on PM. Instead, group discussions involved negotiating how to weigh different considerations as they moved from individual preferences to collective action. This pattern reflects dynamics observed in deliberative settings in PM and health governance, where reasoning develops through dialogue under conditions of uncertainty and competing obligations [16, 17]. Understanding this deliberative dynamic requires positioning SPARK relative to existing educational and engagement formats, which differ in both ambition and design.

SPARK offers a unique blend of traditional ethics teaching, problem-based learning (PBL), team-based learning (TBL), and public deliberation, without fully adhering to a single approach. While it shares with PBL an emphasis on scenario-based discussions, it does not pursue problem resolution and skills assessment [12]. Compared to TBL, SPARK does not rely on advance preparation, readiness assurance testing, or structured peer evaluation. Instead, it uses open-ended scenarios and shared prompts to scaffold in-session deliberation, allowing a small number of facilitators to support large cohorts. In contrast to large-scale public engagement initiatives designed to inform policy and support institutional decision-making, SPARK adapts deliberative principles to a small-scale, time-limited educational setting [18]. It is therefore best understood as a complementary discussion format rather than a substitute for more intensive pedagogical approaches. A more detailed comparison is provided in Supplementary material S7.

The focus on feasibility and accessibility entails important trade-offs. SPARK’s flexible and open structure can lead to variations in discussions among different groups, influenced by facilitation styles and group dynamics. At the same time, predefined cards and prompts support engagement while also shaping the focus of deliberation. Together, the design features reflect a deliberate balance between scalability and pedagogical depth, highlighting SPARK’s strengths in enabling accessible, large-scale participation while also defining the boundaries of what it can achieve within constrained educational settings. This positioning clarifies the intended scope of SPARK as a deliberative activity and provides the basis for considering its practical use and study limitations.

### Practical implementation considerations

SPARK can be adapted for diverse educational and professional groups based on topics and learning goals. In medical education, the format may be particularly valuable early in training, where it can introduce students to the ethical, societal, and systemic dimensions of emerging technologies before clinical routines take precedence. It can also remain valuable in later clinical years, where previous patient contact can deepen discussions through hands-on experience and real-world insights.

Beyond medical training, SPARK is relevant to students in computational biology, biotechnology, data science, and related fields. It may be valuable at later stages of study, when students have developed technical skills to critically engage with the implications of innovation. The format could also be adapted for researchers, healthcare professionals, patient groups, and organisations seeking structured dialogue on technological developments.

The learning outcomes depend on context and facilitation. In education, it promotes awareness of controversial applications, analysis of ethical and societal tensions, and balanced discussion of issues. Rather than replacing knowledge-intensive or performance-based methods, SPARK complements reflection, deliberation, and interdisciplinary exchange.

Broader implementation depends on suitable materials. Developing concise, balanced, and accessible cards on complex topics requires an initial investment of time and topic familiarity, especially during the first design cycle. However, the development burden can be justified by its reuse potential: once a core deck is developed, materials can be reused, adapted, and updated incrementally. In practice, content development benefits from iterative reviews with subject experts and colleagues outside the field to ensure accuracy and clarity. Feedback from students can also help refine materials. To support broader adoption, printable materials, templates, and resources are available in an online repository (see Availability of data and materials), which may further reduce preparation demands across institutions.

Like other discussion-based formats, facilitation plays a key role in how SPARK is experienced across groups. The facilitation approach does not require specialised expertise in PM or advanced teaching credentials. Instead, it benefits from facilitators who can promote wide participation, maintain focus on the topic, encourage participants to share their reasoning, and manage disagreements respectfully within the allotted time. Support for workshop delivery can include a short facilitator orientation, written instructions, example prompts, and co-facilitation when possible. Ultimately, successful implementation relies more on clear structure and basic moderation skills than on a specific facilitator profile.

### Limitations

Several limitations should be noted when interpreting SPARK findings. The study comprised two workshop sessions with students in a specific educational context, which limits generalisability to other settings. Participants were early in their training and not yet embedded in clinical, research, or policy-making roles, and discussions took place within a Norwegian context characterised by strong public-sector involvement in healthcare and social welfare. Furthermore, SPARK was a short-term initiative, and the analysis does not determine whether the observed discussion patterns persist beyond the workshop or affect future practice. In addition, the study examines the integrated format of SPARK rather than isolating the effects of individual design elements. Accordingly, the findings illustrate how structured deliberation shapes discussion processes within a specific setting, rather than demonstrating learning outcomes or attitudinal change.

These considerations open avenues for future research, including applying SPARK with participants at various career stages and across different institutional and policy contexts, to explore how deliberative priorities and collective decision-making vary across settings. Nonetheless, our findings underscore the significance of simple, discussion-oriented formats for exploring the societal dimensions of emerging medical technologies within traditional educational frameworks. This is especially crucial in contexts involving AI-supported PM, where complex questions beyond clinical decision-making arise, yet opportunities for collective reflection are limited in conventional teaching routines.

## Conclusions

Precision medicine is often presented through its technical promise, yet its ethical and societal trade-offs also require attention. In these workshops, students moved beyond primarily technical framings of precision medicine to also consider broader collective and value-based concerns. Our findings suggest that formats such as SPARK may complement ethics education by providing practical opportunities to engage with these ethical and societal dimensions.

## Data Availability

The anonymised datasets generated and analysed during the current study are available in the repository SPARK: Supplementary Materials and Workshop Data figshare repository, DOI link: 10.6084/m9.figshare.31390638.

## Declarations

### Use of Generative Artificial Intelligence (AI)

During manuscript preparation, the authors used ChatGPT (OpenAI, GPT-4.5) and Grammarly for language refinement, structural editing, and improvement of clarity and readability. The tools were used to support phrasing and conciseness. No generative AI tools were used for data analysis, result interpretation, or the generation of original scientific content. All conceptual development, methodological design, analysis, and final editorial decisions were performed by the authors, who take full responsibility for the content of the manuscript.

## Abbreviations

AI: Artificial intelligence
PM: Precision medicine
PBL: Problem-based learning
TBL: Team-based learning
